# *trans*-Dichloridobis(2,4-dimethyl­aniline-κ*N*)palladium(II)

**DOI:** 10.1107/S1600536812015279

**Published:** 2012-04-13

**Authors:** Yong Pan, Hai-Fu Guo, De-Yun Ma, Kuan Lu

**Affiliations:** a College of Chemistry and Chemical Engineering, Zhaoqing University, Zhaoqing 526061, People’s Republic of China; b College of Chemical Engineering, Inner Mongolia University of Technology, Inner Mongolia 010051, People’s Republic of China

## Abstract

In the title compound, [PdCl_2_(C_8_H_11_N)_2_], the Pd^II^ atom is located on a crystallographic inversion center and adopts a square-planar coordination geometry, with pairs of equivalent ligands in *trans* positions. In the crystal, adjacent mol­ecules are linked with each other through weak N—H⋯Cl hydrogen bonds and π–π stacking inter­actions between the phenyl rings [shortest centroid–centroid distance = 3.720 (2) Å], leading to the formation of layers parallel to the *a*-axis direction.

## Related literature

For general background to the application of palladium compounds in homogeneous and heterogeneous catalysis, see: Padmanabhan *et al.* (1985 ▶); Hartley (1973 ▶). For related structures, see: Newkome *et al.* (1982 ▶); Chen *et al.* (2002 ▶).
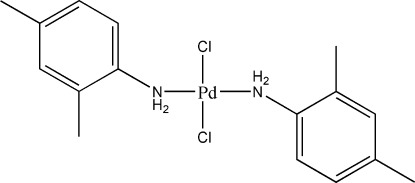


## Experimental

### 

#### Crystal data

[PdCl_2_(C_8_H_11_N)_2_]
*M* *_r_* = 419.66
Monoclinic, 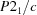

*a* = 14.315 (6) Å
*b* = 8.081 (3) Å
*c* = 7.420 (3) Å
β = 104.705 (7)°
*V* = 830.3 (6) Å^3^
*Z* = 2
Mo *K*α radiation
μ = 1.43 mm^−1^
*T* = 246 K
0.30 × 0.28 × 0.22 mm

#### Data collection

Bruker APEXII area-detector diffractometer
Absorption correction: multi-scan (*SADABS*; Sheldrick, 1996 ▶) *T*_min_ = 0.669, *T*_max_ = 0.740
4058 measured reflections
1485 independent reflections
1226 reflections with *I* > 2σ(*I*)
*R*_int_ = 0.027

#### Refinement

*R*[*F* ^2^ > 2σ(*F* ^2^)] = 0.029
*wR*(*F* ^2^) = 0.076
*S* = 1.06
1485 reflections
99 parameters
H-atom parameters constrained
Δρ_max_ = 1.65 e Å^−3^
Δρ_min_ = −0.66 e Å^−3^

### 

Data collection: *APEX2* (Bruker, 2004) ▶; cell refinement: *SAINT* (Bruker, 2004) ▶; data reduction: *SAINT*; program(s) used to solve structure: *SHELXS97* (Sheldrick, 2008 ▶); program(s) used to refine structure: *SHELXL97* (Sheldrick, 2008 ▶); molecular graphics: *XP* in *SHELXTL* (Sheldrick, 2008 ▶); software used to prepare material for publication: *SHELXL97*.

## Supplementary Material

Crystal structure: contains datablock(s) I, global. DOI: 10.1107/S1600536812015279/zl2469sup1.cif

Structure factors: contains datablock(s) I. DOI: 10.1107/S1600536812015279/zl2469Isup2.hkl

Additional supplementary materials:  crystallographic information; 3D view; checkCIF report

## Figures and Tables

**Table 1:** Hydrogen-bond geometry (Å, °)

*D*—H⋯*A*	*D*—H	H⋯*A*	*D*⋯*A*	*D*—H⋯*A*
N1—H1*B*⋯Cl1^i^	0.91	2.68	3.376 (3)	134
N1—H1*A*⋯Cl1^ii^	0.91	2.39	3.287 (3)	168

Symmetry codes: (i) 
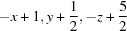
; (ii) 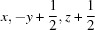.

**Fig. 1.**
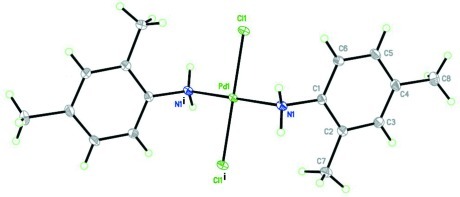
The molecular structure of the title compound, showing 30% probability displacement ellipsoids. Symmetry code: (i) 1 - x, 1 - y, 2 - z.

**Fig. 2.**
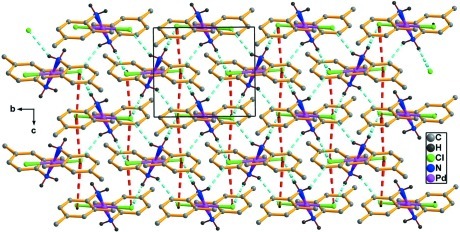
View of one of the two-dimensional layers of the title compound. The intermolecular hydrogen bonds and π–π stacking interactions are shown as turquiose and red dashed lines, respectively. H atoms not involved in hydrogen bonds have been omitted for clarity.

## supplementary crystallographic information

### Comment

Palladium compounds have attracted much attention due to their application in homogeneous and heterogeneous catalyses (Padmanabhan *et al.* 1985). Some dramatic results in homogeneous catalysis of reactions of organic compounds, particularly the successful commercial exploitation of the Wacker one stage process for the homogeneous catalytic oxidation of ethylene to acetaldehyde in the presence of palladium (II) chloride (Hartley 1973), have contributed to this interest. In this paper we report crystallization of the title compound, a new palladium(II) complex obtained by the reaction of 2,4-dimethylaniline with palladium chloride in ethanol. As illustrated in Fig.1, the Pd^II^ atom exhibits a square-planar coordination sphere, defined by two N atoms from two 2,4-dimethylaniline and two chloride atoms. The molecule adopts the *trans* configuration in the solid state. The bond distances of Pd—N (2.055 (2)) and Pd—Cl (2.293 (3) Å) are comparable with the values found in related complexes (Newkome *et al.* 1982; Chen *et al.* 2002). The dihedral angle between the plane of the phenyl ring and the square plane around Pd is 63.03 (1) °. In the crystal structure, intermolecular N—H···Cl hydrogen bonding interactions involving the amino groups and chlorine anions (Table 1) and π–π stacking interactions (centroid-centroid distance = 3.720 (2) Å) occurring between neighboring phenyl rings of centrosymmetrically related complexes form a layer network running parallel to the *a* axis (Fig. 2).

### Experimental

A mixture of palladium chloride (0.1 mmol, 0.018 g) and 2,4-dimethylaniline (0.2 mmol, 0.024 g) in 12 ml of anhydrous ethanol was sealed in an autoclave equipped with a Teflon liner (25 ml) and then heated at 353 K for 1 day. Yellow crystals were obtained by slow evaporation of the solvent at room temperature (0.093 g, 45%). IR (KBr pellet) (cm^-1^): 3452(*s*), 3023(*m*), 2928(*m*), 1619(*s*), 1582(*s*), 1556(*m*), 1488(*s*), 1452(*m*), 1383(*s*), 1283(w), 1231(w), 1184(w), 1143(*m*), 1106(*s*), 1053(*m*), 979(w), 954(w), 891(*m*), 817(*s*), 738(*m*), 607(w), 575(*m*), 466(*m*), 424(*m*).

### Refinement

All H atoms were positioned geometrically and refined using a riding model with the distances of 0.97 Å for methyl groups with *U*_iso_(H) = 1.5*U*_eq_(C) and 0.94 Å for phenyl groups with *U*_iso_(H) = 1.2*U*_eq_(C), respectively. H atoms bonded to N atoms were placed at calculated positions and refined with distance constraints of N—H = 0.91 Å, and with *U*_iso_(H) = 1.2 *U*_eq_(N). The hightest residual electron density peak is located 0.93 Å from Pd1 and the deepest hole is located 0.95 Å from Pd1.

### Crystal data

**Table:**

[PdCl_2_(C_8_H_11_N)_2_]	*F*(000) = 424
*M**_r_* = 419.66	*D*_x_ = 1.679 Mg m^−^^3^
Monoclinic, *P*2_1_/*c*	Mo *K*α radiation, λ = 0.71073 Å
Hall symbol: -P 2ybc	Cell parameters from 5300 reflections
*a* = 14.315 (6) Å	θ = 1.3–28.0°
*b* = 8.081 (3) Å	µ = 1.43 mm^−^^1^
*c* = 7.420 (3) Å	*T* = 246 K
β = 104.705 (7)°	Block, yellow
*V* = 830.3 (6) Å^3^	0.30 × 0.28 × 0.22 mm
*Z* = 2	

### Data collection

**Table:**

Bruker APEXII area-detector diffractometer	1485 independent reflections
Radiation source: fine-focus sealed tube	1226 reflections with *I* > 2σ(*I*)
Graphite monochromator	*R*_int_ = 0.027
φ and ω scan	θ_max_ = 25.2°, θ_min_ = 2.9°
Absorption correction: multi-scan (*SADABS*; Sheldrick, 1996)	*h* = −17→8
*T*_min_ = 0.669, *T*_max_ = 0.740	*k* = −9→9
4058 measured reflections	*l* = −8→8

### Refinement

**Table:**

Refinement on *F*^2^	Primary atom site location: structure-invariant direct methods
Least-squares matrix: full	Secondary atom site location: difference Fourier map
*R*[*F*^2^ > 2σ(*F*^2^)] = 0.029	Hydrogen site location: inferred from neighbouring sites
*wR*(*F*^2^) = 0.076	H-atom parameters constrained
*S* = 1.06	*w* = 1/[σ^2^(*F*_o_^2^) + (0.0402*P*)^2^ + 0.6953*P*] where *P* = (*F*_o_^2^ + 2*F*_c_^2^)/3
1485 reflections	(Δ/σ)_max_ < 0.001
99 parameters	Δρ_max_ = 1.65 e Å^−^^3^
0 restraints	Δρ_min_ = −0.66 e Å^−^^3^

### Special details

**Table:**

Geometry. All e.s.d.'s (except the e.s.d. in the dihedral angle between two l.s. planes) are estimated using the full covariance matrix. The cell e.s.d.'s are taken into account individually in the estimation of e.s.d.'s in distances, angles and torsion angles; correlations between e.s.d.'s in cell parameters are only used when they are defined by crystal symmetry. An approximate (isotropic) treatment of cell e.s.d.'s is used for estimating e.s.d.'s involving l.s. planes.
Refinement. Refinement of *F*^2^ against ALL reflections. The weighted *R*-factor *wR* and goodness of fit *S* are based on *F*^2^, conventional *R*-factors *R* are based on *F*, with *F* set to zero for negative *F*^2^. The threshold expression of *F*^2^ > σ(*F*^2^) is used only for calculating *R*-factors(gt) *etc*. and is not relevant to the choice of reflections for refinement. *R*-factors based on *F*^2^ are statistically about twice as large as those based on *F*, and *R*- factors based on ALL data will be even larger.

### Fractional atomic coordinates and isotropic or equivalent isotropic displacement parameters (Å^2^)

**Table:**

	*x*	*y*	*z*	*U*_iso_*/*U*_eq_	
C1	0.3227 (3)	0.3340 (4)	1.0653 (5)	0.0177 (7)	
C2	0.2358 (3)	0.4055 (4)	0.9726 (5)	0.0238 (8)	
C3	0.1603 (3)	0.3005 (4)	0.8916 (6)	0.0270 (9)	
H3	0.1009	0.3471	0.8282	0.032*	
C4	0.1686 (3)	0.1303 (4)	0.9001 (6)	0.0257 (9)	
C5	0.2558 (3)	0.0642 (4)	0.9939 (5)	0.0242 (8)	
H5	0.2633	−0.0514	1.0016	0.029*	
C6	0.3321 (3)	0.1643 (4)	1.0765 (5)	0.0203 (8)	
H6	0.3911	0.1170	1.1410	0.024*	
C7	0.2219 (3)	0.5877 (5)	0.9583 (7)	0.0365 (11)	
H7A	0.2382	0.6358	1.0822	0.055*	
H7B	0.1550	0.6121	0.8973	0.055*	
H7C	0.2633	0.6342	0.8862	0.055*	
C8	0.0835 (3)	0.0239 (5)	0.8099 (7)	0.0385 (11)	
H8A	0.0807	0.0104	0.6787	0.058*	
H8B	0.0246	0.0762	0.8228	0.058*	
H8C	0.0903	−0.0837	0.8698	0.058*	
Cl1	0.55490 (6)	0.23246 (9)	1.01658 (12)	0.0208 (2)	
N1	0.4044 (2)	0.4353 (3)	1.1530 (4)	0.0181 (6)	
H1A	0.4382	0.3811	1.2567	0.022*	
H1B	0.3815	0.5302	1.1918	0.022*	
Pd1	0.5000	0.5000	1.0000	0.01533 (14)	

### Atomic displacement parameters (Å^2^)

**Table:**

	*U*^11^	*U*^22^	*U*^33^	*U*^12^	*U*^13^	*U*^23^
C1	0.0191 (18)	0.0188 (16)	0.0165 (18)	−0.0024 (14)	0.0070 (15)	−0.0018 (14)
C2	0.022 (2)	0.0193 (18)	0.029 (2)	−0.0006 (16)	0.0055 (17)	0.0025 (15)
C3	0.0184 (19)	0.0231 (19)	0.037 (2)	0.0011 (16)	0.0018 (17)	0.0026 (17)
C4	0.025 (2)	0.0218 (18)	0.031 (2)	−0.0067 (17)	0.0068 (18)	−0.0052 (15)
C5	0.029 (2)	0.0158 (16)	0.030 (2)	−0.0039 (16)	0.0114 (18)	−0.0018 (15)
C6	0.022 (2)	0.0186 (16)	0.0195 (19)	0.0013 (15)	0.0047 (16)	0.0025 (14)
C7	0.026 (2)	0.0183 (19)	0.061 (3)	0.0018 (17)	0.003 (2)	0.0023 (19)
C8	0.026 (2)	0.031 (2)	0.055 (3)	−0.0114 (19)	0.006 (2)	−0.008 (2)
Cl1	0.0274 (5)	0.0131 (4)	0.0224 (4)	0.0013 (4)	0.0071 (4)	0.0008 (3)
N1	0.0223 (16)	0.0142 (13)	0.0168 (15)	−0.0007 (12)	0.0033 (13)	−0.0007 (12)
Pd1	0.0192 (2)	0.0107 (2)	0.0155 (2)	−0.00095 (15)	0.00342 (15)	−0.00031 (14)

### Geometric parameters (Å, º)

**Table:**

C1—C6	1.378 (5)	C7—H7A	0.9700
C1—C2	1.385 (5)	C7—H7B	0.9700
C1—N1	1.441 (4)	C7—H7C	0.9700
C2—C3	1.385 (5)	C8—H8A	0.9700
C2—C7	1.486 (5)	C8—H8B	0.9700
C3—C4	1.381 (5)	C8—H8C	0.9700
C3—H3	0.9400	Cl1—Pd1	2.2930 (11)
C4—C5	1.373 (5)	N1—Pd1	2.055 (3)
C4—C8	1.503 (5)	N1—H1A	0.9100
C5—C6	1.372 (5)	N1—H1B	0.9100
C5—H5	0.9400	Pd1—N1^i^	2.055 (3)
C6—H6	0.9400	Pd1—Cl1^i^	2.2930 (11)
			
C6—C1—C2	120.4 (3)	C2—C7—H7C	109.5
C6—C1—N1	118.8 (3)	H7A—C7—H7C	109.5
C2—C1—N1	120.8 (3)	H7B—C7—H7C	109.5
C1—C2—C3	117.6 (3)	C4—C8—H8A	109.5
C1—C2—C7	122.4 (3)	C4—C8—H8B	109.5
C3—C2—C7	120.0 (3)	H8A—C8—H8B	109.5
C4—C3—C2	122.8 (4)	C4—C8—H8C	109.5
C4—C3—H3	118.6	H8A—C8—H8C	109.5
C2—C3—H3	118.6	H8B—C8—H8C	109.5
C5—C4—C3	117.9 (3)	C1—N1—Pd1	118.3 (2)
C5—C4—C8	122.2 (3)	C1—N1—H1A	107.7
C3—C4—C8	120.0 (4)	Pd1—N1—H1A	107.7
C4—C5—C6	121.0 (3)	C1—N1—H1B	107.7
C4—C5—H5	119.5	Pd1—N1—H1B	107.7
C6—C5—H5	119.5	H1A—N1—H1B	107.1
C5—C6—C1	120.4 (3)	N1—Pd1—N1^i^	180.0
C5—C6—H6	119.8	N1—Pd1—Cl1	89.86 (8)
C1—C6—H6	119.8	N1^i^—Pd1—Cl1	90.14 (8)
C2—C7—H7A	109.5	N1—Pd1—Cl1^i^	90.14 (8)
C2—C7—H7B	109.5	N1^i^—Pd1—Cl1^i^	89.86 (8)
H7A—C7—H7B	109.5	Cl1—Pd1—Cl1^i^	180.0

Symmetry code: (i) −*x*+1, −*y*+1, −*z*+2.

### Hydrogen-bond geometry (Å, º)

**Table:**

*D*—H···*A*	*D*—H	H···*A*	*D*···*A*	*D*—H···*A*
N1—H1*B*···Cl1^ii^	0.91	2.68	3.376 (3)	134
N1—H1*A*···Cl1^iii^	0.91	2.39	3.287 (3)	168

Symmetry codes: (ii) −*x*+1, *y*+1/2, −*z*+5/2; (iii) *x*, −*y*+1/2, *z*+1/2.

## References

[bb7] Bruker (2004). *APEX2* and *SAINT* Bruker AXS Inc., Madison, Wisconsin, USA.

[bb1] Chen, Y. B., Li, Z. J., Qin, Y. Y., Kang, Y., Wu, L. & Yao, Y. G. (2002). *Chin. J. Struct. Chem.* **21**, 530–532.

[bb2] Hartley, F. R. (1973). In *The Chemistry of Platinum and Palladium* New York: John Wiley and Sons.

[bb3] Newkome, G. R., Fronczek, F. R., Grupta, V. K., Puckett, W. E., Pantaleo, D. C. & Kiefer, G. E. (1982). *J. Am. Chem. Soc.* **104**, 1782–1783.

[bb4] Padmanabhan, V. M., Patel, R. P. & Ranganathan, T. N. (1985). *Acta Cryst.* C**41**, 1305–1307.

[bb5] Sheldrick, G. M. (1996). *SADABS* University of Göttingen, Germany.

[bb6] Sheldrick, G. M. (2008). *Acta Cryst.* A**64**, 112–122. 10.1107/S0108767307043930 18156677

